# Encounter Decision Aid vs. Clinical Decision Support or Usual Care to Support Patient-Centered Treatment Decisions in Osteoporosis: The Osteoporosis Choice Randomized Trial II

**DOI:** 10.1371/journal.pone.0128063

**Published:** 2015-05-26

**Authors:** Annie LeBlanc, Amy T. Wang, Kirk Wyatt, Megan E. Branda, Nilay D. Shah, Holly Van Houten, Laurie Pencille, Robert Wermers, Victor M. Montori

**Affiliations:** 1 Department of Health Sciences Research, Division of Health Care Policy and Research, Mayo Clinic, Rochester, MN, United States of America; 2 Knowledge and Evaluation Research Unit, Mayo Clinic, Rochester, MN, United States of America; 3 Robert D. and Patricia E. Kern Mayo Clinic Center for the Science of Healthcare Delivery, Mayo Clinic, Rochester, MN, United States of America; 4 Division of General Internal Medicine, Los Angeles Biomedical Research Institute at Harbor-UCLA Medical Center, Torrance, CA, United States of America; 5 Department of Medicine, Division of Pediatrics, Mayo Clinic, Rochester, MN, United States of America; 6 Department of Health Sciences Research, Division of Biomedical Statistics and Informatics, Mayo Clinic, Rochester, MN, United States of America; 7 Department of Medicine, Division of Endocrinology, Mayo Clinic, Rochester, MN, United States of America; Garvan Institute of Medical Research, AUSTRALIA

## Abstract

**Purpose:**

Osteoporosis Choice, an encounter decision aid, can engage patients and clinicians in shared decision making about osteoporosis treatment. Its effectiveness compared to the routine provision to clinicians of the patient’s estimated risk of fracture using the FRAX calculator is unknown.

**Methods:**

Patient-level, randomized, three-arm trial enrolling women over 50 with osteopenia or osteoporosis eligible for treatment with bisphosphonates, where the use of Osteoporosis Choice was compared to FRAX only and to usual care to determine impact on patient knowledge, decisional conflict, involvement in the decision-making process, decision to start and adherence to bisphosphonates.

**Results:**

We enrolled 79 women in the three arms. Because FRAX estimation alone and usual care produced similar results, we grouped them for analysis. Compared to these, use of Osteoporosis Choice increased patient knowledge (median score 6 vs. 4, p = .01), improved understanding of fracture risk and risk reduction with bisphosphonates (p = .01 and p<.0001, respectively), had no effect on decision conflict, and increased patient engagement in the decision making process (OPTION scores 57% vs. 43%, p = .001). Encounters with the decision aid were 0.8 minutes longer (range: 33 minutes shorter to 3.0 minutes longer). There were twice as many patients receiving and filling prescriptions in the decision aid arm (83% vs. 40%, p = .07); medication adherence at 6 months was no different across arms.

**Conclusion:**

Supporting both patients and clinicians during the clinical encounter with the Osteoporosis Choice decision aid efficiently improves treatment decision making when compared to usual care with or without clinical decision support with FRAX results.

**Trial Registration:**

clinical trials.gov NCT00949611

## Introduction

Several interventions reduce the risk of fragility fractures in at-risk individuals[1]. Apart from lifestyle modification, calcium and vitamin D, bisphosphonates are the most commonly recommended intervention given their known efficacy, ease of use, relatively low cost, and short-term safety; however the value of this therapy is often reduced by poor patient adherence[1,2,3,4]. Our previous work revealed that patient preferences play a large role in taking up or rejecting bisphosphonates, even among women at high risk of osteoporotic fractures[5,6].

Recognition of the role patient preferences play in taking up bisphosphonates and in adhering to therapy has led to efforts to engage patients in the decision to take bisphosphonates. The opportunity to engage patients in this decision has improved as a result of the 2008 World Health Organization (WHO)’ s FRAX calculator[7,8]. This calculator, available online and integrated in some electronic medical records, should improve the ability of patients and clinicians to discuss treatment options, moving from technically challenging concepts such as bone mineral density or T-scores into the use of estimated ten-year risk of bone fragility fractures. Using the FRAX calculator to determine an individual’s risk of fracture and robust information about bisphosphonates’ efficacy in reducing this risk, our group developed an encounter decision aid in 2008, the Osteoporosis Choice decision aid, to facilitate shared decision making during the clinical encounter[9].

We conducted a pilot, randomized trial of 100 patients, evaluating the effect of the Osteoporosis Choice decision aid[5]. This trial found that the use of the tool improved the quality of clinical decisions about bisphosphonate therapy by improving knowledge transfer and patient involvement, did not affect start rates, and may have improved adherence[5]. However, by the time of completion of the study, in addition to its online availability, the FRAX calculator had been endorsed by U.S clinical practice guidelines and was making its way into the reporting software for bone densitometry (serving as a clinical decision support tool), thus fast becoming commonplace in primary care practice. Thus, we wondered to what extent clinician use of the FRAX would be sufficient to inform and engage patients in their care. We were also interested in determining the incremental value, if any, of the Osteoporosis Choice decision aid.

Consequently, we sought to determine the effect of the Osteoporosis Choice decision aid compared to usual care with and without the FRAX fracture risk calculator on patient knowledge, decisional conflict, involvement in the decision making process, decision to start medication, adherence to bisphosphonates, acceptability, and satisfaction with the decision-making process.

## Materials and Methods

The protocol for this trial and supporting CONSORT checklist are available as supporting information; see S1 CONSORT Checklist and S1 Protocol.

### Study design and setting

We conducted a multicenter, patient level, randomized trial in the midst of the usual flow of routine primary care practices. The study was originally designed to recruit patients and randomize them to one of two arms: (i) use of the Osteoporosis Choice decision aid during the clinical encounter (Decision Aid), or (ii) provision of the patient’s FRAX estimate only to the clinician (FRAX). After 5 months of enrollment, we added a usual care arm—in which the FRAX score was not made available during encounters—in order to offer a direct comparison against all major practice patterns at that time.

The Mayo Clinic Institutional Review Board approved the study protocol and all study procedures on March 4 2009, when the trial was submitted for registration at clinicaltrials.gov. Unforeseen delays in the release of the registration record due to errors and corrections created an unexpected lapse between approval and registration. The first patient was enrolled in May 2009, and clinical trials.gov released the registration record (Identifier: NCT00949611) on July 28, 2009. Patients and clinicians gave written informed consent.

### Study population

Participating practices (Family Medicine, Preventive Medicine, Primary Care Internal Medicine and General Internal Medicine) were all affiliated to the Mayo Clinic (Rochester, Minnesota, USA). Clinicians (i.e., physicians, nurse practitioners) from participating primary care practices were eligible to take part in the study if they provided care for patients with osteopenia or osteoporosis. English-speaking women from participating practices were eligible if they were over 50, with a diagnosis of osteopenia or osteoporosis, were not taking bisphosphonates or other prescription medications to treat their condition, were identified by their clinician as potentially eligible for bisphosphonates, were available for a six month follow up after randomization, and had no major learning barriers.

Eligible patients were identified through lists of upcoming bone mineral density evaluations and from participating clinicians’ appointment calendars, and approached by study coordinators at the time of their appointment.

### Intervention and usual care

**Fig 1:**
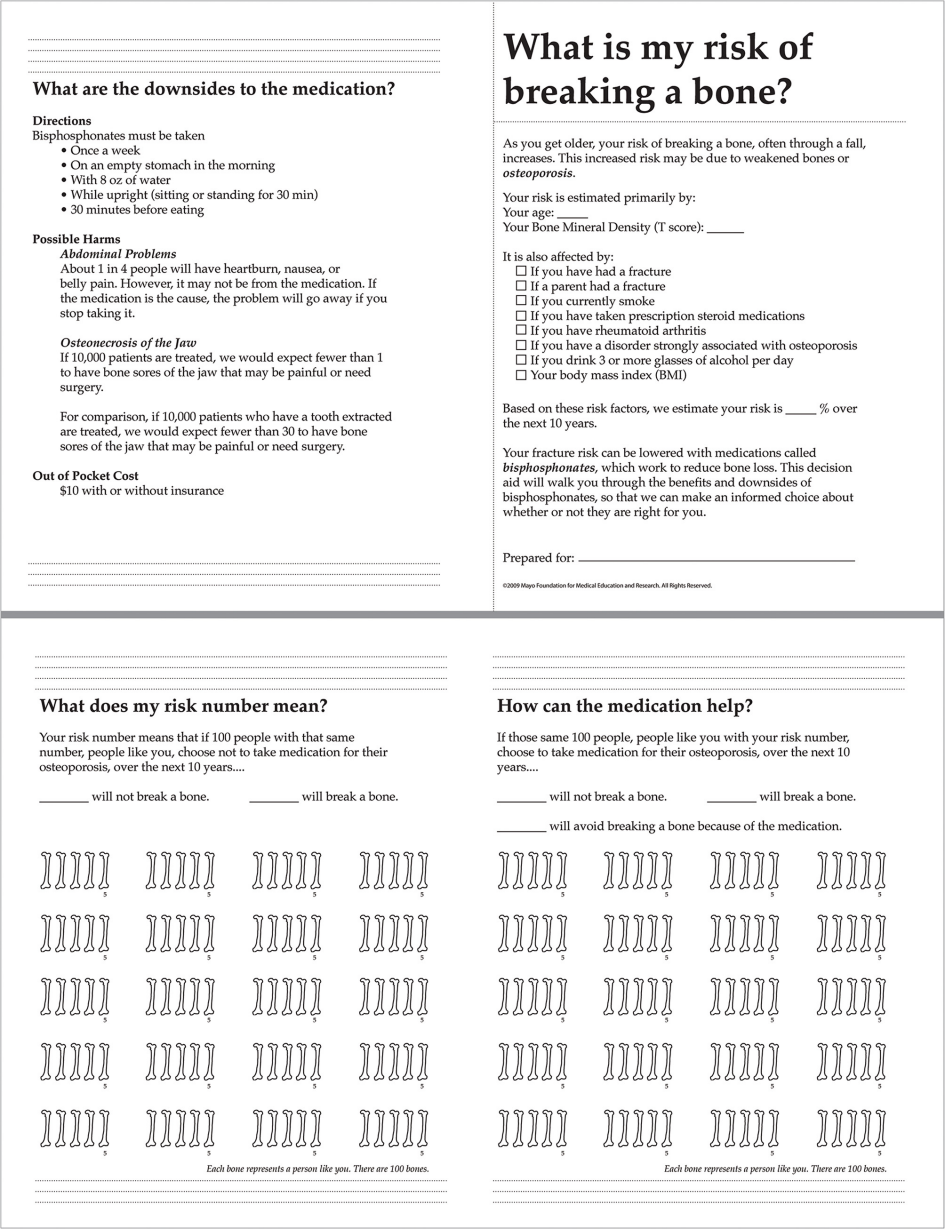
The Osteoporosis Choice Decision Aid (unfilled).

The intervention in the first arm (Decision Aid) consisted of the use of the Osteoporosis Choice decision aid by the clinician and patient during the clinical encounter (**Fig 1**). The decision aid included (a) the individualized 10-year risk of having a bone fracture (estimated using the FRAX calculator) with and without use of bisphosphonates (i.e., showing the absolute reduction with bisphosphonates) represented using an evidence-based pictograph and assuming a treatment-related reduction in overall fractures of 40%[10]; and (b) potential harms and other downsides of using bisphosphonates. The study coordinator prepared each decision aid according to each patient’s characteristics prior to the clinical encounter (See **S1 Fig** for an example of a filled decision aid). Patients and clinicians were to review the decision aid, deliberate about whether to start bisphosphonates, and make a decision together at that time or at a later time. The intervention in the second arm (FRAX) consisted of giving clinicians a copy of the patient’s individualized 10-year risk of having a bone fracture estimated using the FRAX calculator before the visit for use during the clinical encounter. In the third arm (Usual Care), clinicians discussed risk of fractures and treatment as usual without any research-related intervention. No specific guidance was provided to support decisions about non-pharmacological interventions to reduce falls and fractures in any of the three arms.

### Randomization, allocation concealment, and blinding

Patients were the unit of randomization and were allocated using a computer-generated sequence that randomized them 1:1:1 in a concealed fashion. Stratification factors were baseline fracture risk categorization (<20% 10-year risk not on any osteoporosis medication other than bisphosphonates, <20% with a concurrent osteoporosis medication other than bisphosphonates, >20%), practice type (local vs. referral population) and clinician’s likelihood of prescribing medication (low vs. high) assessed from a scenario-based question at the start of the study. The balance of stratification factors was actively monitored and no imbalance was noted across the three arms.

After randomization, only data analysts remained blind to allocation. Patients and clinicians were aware of the overall objective, presented as improvement in communication between patients and clinicians during the clinical encounter, but remained blinded to the specific aims. Research staff at Mayo Clinic monitored patients’ medical records, administered follow-up surveys, and requested pharmacy records.

Possible contamination at the clinician level (i.e. clinician who, having used the decision aid with a prior patient, recreates elements of the decision aid with a subsequent patient allocated to receive FRAX alone or usual care) was monitored by a detailed review of the available video recorded encounters (see *Fidelity* assessment below).

### Data collection and outcomes

Patients and clinicians completed surveys immediately after their clinical encounters, in addition to providing demographic information upon enrollment into the study.

Clinical encounters were video recorded when both the patient and the clinician consented. Medical records were reviewed at patient enrollment and at six months post encounter. Patients completed a telephone follow-up six months later. We also obtained pharmacy records for the period 3 months prior to enrollment to 9 months post enrollment.

We measured patients’ knowledge, decisional conflict, decision to start medication, adherence to medication, involvement in decision making by the clinician, fidelity to intended intervention, acceptability, satisfaction, and quality of life. Decision quality was designated as a secondary outcome.

We assessed patients’ knowledge post-encounter using a 13-item questionnaire that was developed for and used in our previous study, which included questions pertaining to information found within the decision aid (n = 9) and questions regarding overall osteoporosis knowledge (n = 4)[5,9]. We also asked patients to estimate their 10-year risk of breaking a bone at baseline and with the use of bisphosphonates. Patient decisional conflict was assessed post encounter using the Decisional Conflict Scale, the most commonly used outcome measure in decision aid trials[11]. The decision to start bisphosphonates or not was recorded by patient survey immediately after the encounter. We collected patients’ prescription drug and billing data through pharmacy records and assessed adherence to their prescribed medication through 6-month self-reported data and pharmacy records. Medication adherence was evaluated in two ways[12,13]. First, by calculating the percentage of patients who filled their initial prescription (primary adherence). Then, by calculating the percentage of days covered (PDC), defined as the number of days a patient had a supply of each medication divided by the number of days of eligibility of that medication, and the proportion of PDC >80%, for both, those who filled the prescription and all who received a prescription (secondary adherence). We considered medications within the drug class of bisphosphonates interchangeable.

Patients’ involvement in the decision making process by the clinician was assessed by reviewing video-recording of encounters using the OPTION scale, a third-person observer scale designed for use in reviewing audio recordings of primary care visits[14]. We also assessed, by reviewing the video-recorded encounters, the fidelity with which the decision aid was delivered and used as intended during these encounters using the osteoporosis fidelity checklist[5]. This scale is comprised of 10 items (present/absent scale), and results are presented as the percentage of items present. We assessed patients’ and clinicians’ acceptability of and satisfaction with the decision aid using questions that require them to assess the extent to which they would want for themselves and recommend to others similar decision support as they received/provided during the visit. Patients’ quality of life was measured using the EURO QOL5d health thermometer, which assesses patients current health state on a scale of 0–100[15]. Clinicians’ perspective of patient’s perception of the effectiveness of the decision making process was assessed post-encounter using a modified version of the decisional conflict scale effectiveness subscale[11]. Duration of encounters was estimated based on the recorded time of the clinical encounters[5].

### Sample size

We powered our trial to have adequate precision in the estimation of the comparative effectiveness of the interventions on knowledge and acceptability, recognizing that rates of start, stop and adherence to bisphosphonates may require joint consideration of this and our prior trial. While we estimated enrolling 150 participants (enough to have 80% power to detect a 30% difference in adherence between arms at 6 months, but underpowered to detect smaller yet important differences), funding run out before we could reach that target. On the basis of the findings from our initial Osteoporosis Choice trial[5], in which we reported a mean patient knowledge score of 3.9 in the usual care arm compared to a mean score of 5.7 for the decision aid arm (standard deviation of 2.5) out of 13 questions, we estimate the accrued sample would provide about 80% power to detect a difference of this magnitude in this trial, and a 0.75 standard deviation difference on any continuous outcome (e.g., decisional conflict scale) across two arms, with a two-sided test and an alpha of 0.05.

### Data analysis

We analyzed all patients as randomized, adhering to the intention to treat principle[16]. We present patient and clinician characteristics as counts and frequencies for categorical data and means and 95% CI or medians and interquartile range, as appropriate for data, for continuous outcomes within each of the three arms. The comparison of the arms was conducted using the Chi-square or Fisher’s exact test for categorical outcomes and for continuous outcomes are compared using the t-test or Wilcoxon rank sum test.

## Results

**Fig 2:**
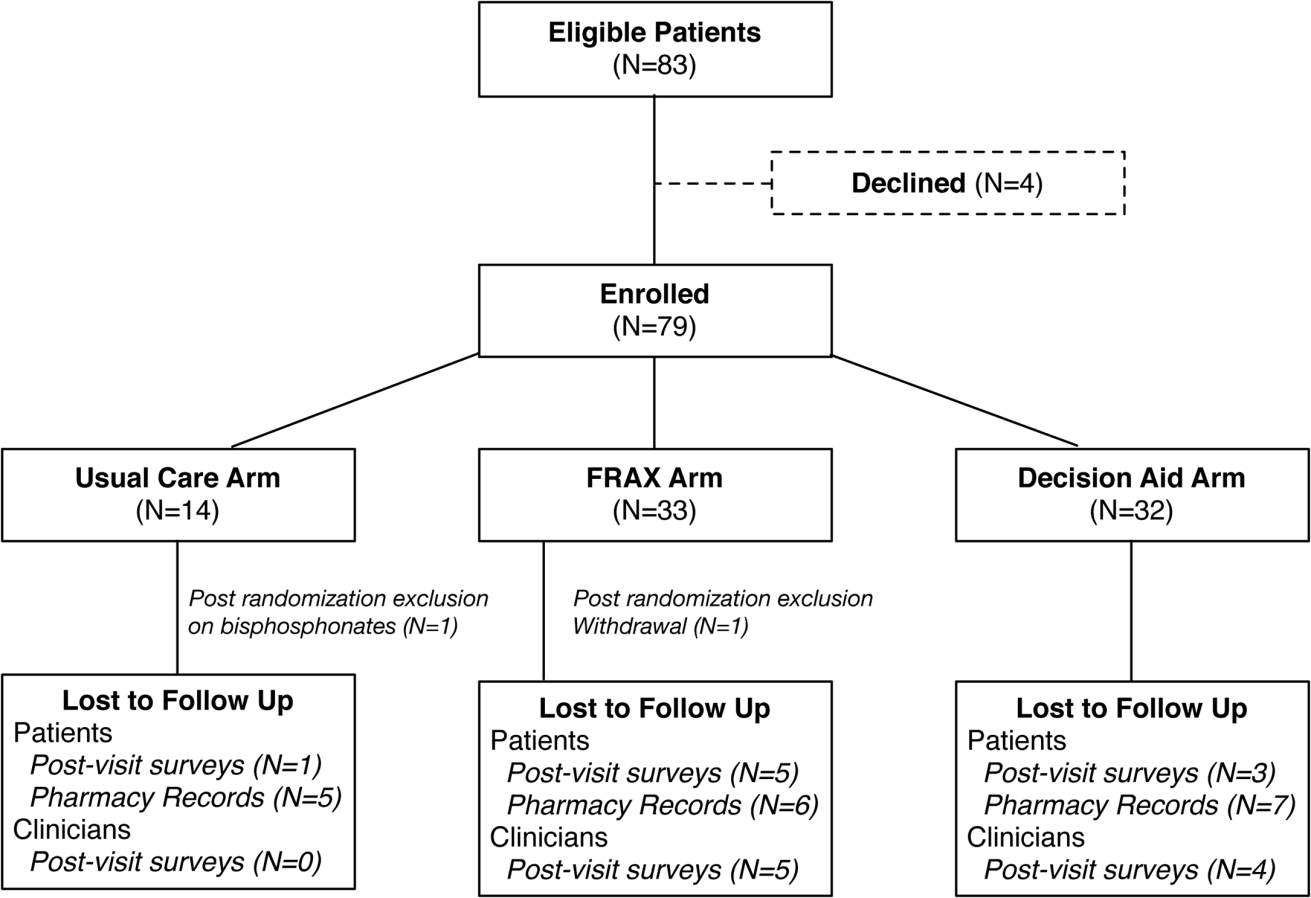
Flow Chart.

We enrolled 41 clinicians and 79 patients between May 2009 and April 2010 (**Fig 2**). The first 33 patients were randomized between one of the two intervention arms only, and the remaining patients were randomly assigned to all three arms. Overall 32 patients were assigned to the Decision Aid arm, 33 to the FRAX arm, and 14 to the Usual Care arm. Two patients were excluded from analysis after enrollment; a patient from the usual care arm was on bisphosphonates at the time of enrollment (participant selection error) and a patient from the FRAX arm withdrew her consent. **Table 1**describes the characteristics of participating clinicians and patients.

**Table 1:** Patient and clinician characteristics.

Characteristics	Decision Aid	FRAX/Usual Care
Patients	N = 32	N = 45
Age, mean(SD), yrs	69 (8)	66 (10)
Body Mass Index, mean(SD)	27 (6)	28 (5)
Education, N(%)^1^		
*High School or Less*	8 (25)	10 (24)
*Some College*	14 (44)	16 (39)
*4 yrs College +*	9 (28)	15 (37)
Income, N(%)^1^		
*<40 000$/ yr*	12 (38)	10 (26)
*40–80 000$/ yr*	5 (16)	15 (38)
*>80 000$/ yr*	8 (25)	14 (36)
Subjective Numeracy, mean (SD)	4.3 (1.0)	4.2 (1.0)
Risk of a bone fracture		
FRAX score, Mean % (SD)	14 (8)	13 (7)
*Low (<10%)*, *N(%)*	10 (31)	21 (47)
*Moderate (11–20%)*, *N(%)*	16 (50)	17 (38)
*High (>20%)*, *N(%)*	6 (19)	7 (15)
**Clinicians**	**N = 22**	**N = 28**
Gender, N(%), female	8 (36)	14 (50)
Type		
Local Primary Care, N(%)	9 (41)	7 (25)
Referral practice, N(%)	13 (59)	21 (75)
Cinician’s likelihood of prescribing medication^2^, mean (SD)	2.8 (1.3)	2.5 (1.5)
Encounters^3^, mean, median(range)	1.5, 1 (1, 4)	1.6, 1 (1, 9)

^1^Values missing for patients

^2^Bias assessment of prescribing rates assessed for stratifying patients

^3^Number of encounters included in the study; SD = Standard deviation

We found no difference between the FRAX and usual care arms, nor were overall results significantly impacted by analyses comparing the three arms versus only two arms (Decision aid arm vs. FRAX/usual care arms together, see **Tables A, B, C, D, and E** in S1 File. Therefore, the results comparing the FRAX arm and the Usual Care arm were combined and all subsequent results are presented as Decision Aid vs. FRAX/Usual Care arm (i.e. different forms of usual care). No significant differences were found when comparing the characteristics of patients in the Decision Aid arm with patients in the FRAX/Usual Care combined arms (results not shown).

We observed a significant increase in patient knowledge in the Decision Aid arm about matters covered in the decision aid, and no difference in knowledge of matters not covered in the decision aid (**Table 2**). More patients reported their risk without and with medication with accuracy in the Decision Aid arm than in the FRAX/Usual Care arm (**Table 2**). Decisional conflict was low for both groups, and was lower in the Decision Aid arm, but no significant difference was found in the overall scale or in its subscales across arms (**Table 2**).

**Table 2:** Patient knowledge and decisional conflict.

	Decision Aid	FRAX/Usual Care	Relative Risk (95% CI)^3^	P-Value
**Knowledge** ^**1**^	**N = 32**	**N = 45**		
Overall (13 items)	7.0 (4.5, 9.0)	5.5 (2.5, 8.0)		0.11
*Tailored knowledge (9 items)*	6.0 (3.5, 6.5)	4.0 (2.0, 5.0)		0.01
*Generic knowledge (4 items)*	1.5 (0, 3.0)	1.5 (0, 3.0)		0.98
**Knowledge of Risk** ^**2**^	**N = 29**	**N = 40**		
Risk without medication	20 (69)	14 (35)	2.0 (1.2, 3.2)	0.01
Post-treatment risk	23 (79)	12 (30)	2.6 (1.6, 4.4)	<.0001
**Decisional Conflict** ^**1**^	**N = 28**	**N = 36**		
Overall	10.9 (1.6, 26.6)	22.7 (7.8, 28.5)		0.18
*Informed subscale*	4.2 (0, 25.0)	20.8 (0, 33.3)		0.14
*Clarity subscale*	16.7 (0, 25.0)	25.0 (8.3, 33.3)		0.25
*Support subscale*	8.3 (0, 25.0)	16.7 (0, 25.0)		0.35
*Certainty subscale*	8.3 (0, 25.0)	25.0 (0, 25.0)		0.30
*Effectiveness subscale*	12.5 (0, 25.0)	18.8 (0, 25.0)		0.15

^1^Median (IQR), Wilcoxon rank sum test p-value

^2^Answered correctly (% Correct), Chi-square test p-value.

^3^Decision Aid is the reference for Relative Risk.

Clinicians prescribed bisphosphonates to 25 (37%) patients (**Table 3**). Although underpowered, we noted that more patients in the Decision Aid arm were prescribed bisphosphonates (41% vs. 27% in FRAX/usual care arm, p = .20) and decided to fill that prescription (83% vs. 40% in the FRAX/Usual Care arm, p = .07, **Table 3**).

**Table 3:** Decision and adherence.

	Decision Aid	FRAX/Usual Care	Relative Risk (95% CI)^3^	P-Value
**Patient decision per survey** ^1^ ^,5^:	**N = 29**	**N = 38**		
Start Bisphosphonates	12 (41)	11 (29)	1.4 (0.7, 2.7)	0.57
Do Not Start	8 (28)	13 (34)		
Undecided/Other	9 (31)	14 (37)		
**Prescription during encounter** ^1^	13 (41)	12 (27)	1.5 (0.8, 2.9)	0.20
**Primary adherence**				
Filled Prescription^1^	10 (83)	4 (40)	2.1 (0.9, 4.6)	0.07^4^
*Record Missing*	1	2		
**Secondary adherence**				
% of days covered out of 180^2^	46.7 (39.2, 46.7)	85 (55.3, 92.6)		0.08

^1^Counts (%),Chi-square test p-value, unless noted otherwise

^2^Median (95% CI), Wilcoxon Rank Sum Test p-value

^3^Decision Aid is the reference of relative risk where ‘Start Bisphosphonates’ is being compared to the combination of ‘Do not start’ and ‘Undecided/Other’

^4^Fisher’s Exact Test p-value

No difference was found in adherence to the medication that was prescribed for both, those who filled their initial prescription [PDC Median 46.7%, IQR (30, 62) for Decision Aid arm vs. 85%, IQR (55.3, 92.6) for FRAX/Usual Care arm, **Table 3**] or for all that were prescribed bisphosphonates [PDC Median (IQR) 46.7%, (7.8, 46.7) for Decision Aid arm vs. 0%, (0, 72.5) for FRAX/Usual Care arm].Only one patient in the Decision Aid arm and 3 in the FRAX/Usual Care arm had PDC >80%.

Thirty-eight patients agreed to be video recorded (Decision Aid = 25 vs. FRAX/Usual Care = 13). Encounters that were recorded did not differ from those not recorded for any patient factors except income. Encounters recorded were more likely to have patients with income > $80,000 per year (49% vs. 19% not recorded, p = .04). We reviewed these videos and found that patient involvement in the decision making process was significantly higher in the Decision Aid arm [OPTION score 57%, 95%CI (50, 64)] compared to the FRAX/Usual Care arm [43%, 95%CI (37, 48), t-test p = 0.001]. We also found that the fidelity with which the decision aid items were covered was high in the Decision Aid arm [67%, 95%CI (63, 78)]; there was no evidence of contamination as clinicians in the FRAX/Usual Care arm covered significantly fewer items [17%, 95%CI (12, 23), t-test p< 0.0001].

Patients were satisfied with all methods of sharing information within the encounter: when asked if they would recommend to other patients the method they received, 86% in the Decision Aid arm said they would compared to 77% in the FRAX/Usual Care arm (p = .52) (**Table 4**). Clinicians also found the decision aid extremely helpful in providing their patients with information (Decision Aid arm = 70% vs. FRAX/Usual Care arm = 35%, p = .01) and would recommend that other clinicians provide information to patients in a similar manner (Decision Aid arm = 74% vs. FRAX/Usual Care arm = 30%, p = <.001), for the decisions about osteoporosis therapy and for any other health related decision (Decision Aid arm = 67% vs. FRAX/Usual Care arm = 41%, p = .04) (**Table 4**). Encounter duration in the FRAX/Usual Care arm had a median of 10.7 minutes and a range of 2.5 to 54.9 minutes, where encounters in the Decision Aid arm had a median duration of 11.5 with a range of 5.4 to 21.4 minutes (median difference 0.8 minutes, range -33.6 to 3.0).

**Table 4:** Patient satisfaction.

	Decision Aid	FRAX/Usual Care	Relative Risk (95% CI)^3^	P-Value
**Patients**	**N = 29**	**N = 37**		
**Satisfaction**				
Amount of information was just right^1^	25 (86)	34 (92)	0.9 (0.8, 1.1)	0.69^4^
Information received was clear^1^	17 (63)	26 (72)	0.9 (0.6, 1.2)	0.43
*Reply missing*	2	1		
Information received was helpful^1^	21 (75)	23 (68)	1.1 (0.8, 1.5)	0.53
*Reply missing*	1	3		
Would recommend method to others^1^	24 (86)	27 (77)	1.1 (0.9, 1.4)	0.52^4^
*Reply missing*	1	2		
**Quality of life**				
EQOL 5D Health Thermometer^2^	85 (80, 95)	85 (73, 90)		0.19
**Clinicians**				
**Satisfaction**	**N = 27**	**N = 40**		
Information was helpful to the patient^1^	19 (70)	14 (35)	2.0 (1.2, 3.2)	0.01
Would recommend method to other providers^1^ ^`^	20 (74)	12 (30)	2.5 (1.5, 4.2)	<.001
Would present information in the same manner for other treatment decisions^1^	18 (67)	16 (41)	1.6 (1.0, 2.6)	0.04
*Reply missing*	0	1		
**Perception of effectiveness (DCS)** ^2^	25 (6, 25)	25 (14, 25)		0.18

^1^Counts (%), Chi-Square test p-value, unless noted otherwise

^2^Median (IQR), Wilcoxon Rank Sum Test p-value

^3^Decision Aid is the reference for relative risk

^4^Fisher’s exact test p-value

## Discussion

### Main findings

We found that using the Osteoporosis Choice decision aid was better than usual care with or without FRAX calculation in improving patient knowledge and patient engagement in deciding whether to start bisphosphonates in at-risk women. Using the decision aid changed the length of the discussion, on average, by less than a minute. Clinicians found the decision aid extremely helpful and would recommend it to others. More patients started a bisphosphonate and filled their prescriptions in the Decision Aid arm compared to the FRAX/Usual Care arm; however this was not statistically significant due in part to the relative small number of actual bisphosphonate starts.

Another important finding was that using the FRAX calculator alone as a clinical decision support tool during the encounter was no different than usual care across all measured parameters. The FRAX calculator provides a specific 10-year risk estimate for fracture, a presumed enhancement over risk unawareness or risk communication inferred from the results of bone mineral density tests. Perhaps the addition of an exact number versus a gestalt range from a clinician is not important or not perceived as different to a patient, or perhaps clinicians did not use the FRAX results to communicate quantitatively with patients. It is also possible that the layout and design of the decision aid better fosters communication of the patient’s individual risk; provision of the FRAX score to the clinician does not necessarily mean that a clinician knows how to use that score in a clinical encounter with a patient or that they will actually do so. The decision aid communicates not just the risk of fracture but also quantifies the potential risk reduction with bisphosphonate therapy. The decision aid also brings various patient important issues (i.e., side effects, cost) to the foreground and serves as an invitation for the patient and clinician to address these. These unique features of the decision aid help to create a conversation centered on what is important to the patient and likely facilitated the increase in patient engagement in the Decision Aid arm compared to the FRAX/Usual Care arm.

Our results are consistent with decision aid trials that show improved knowledge and patient engagement and decreased decision conflict with decision aid use. Cranney et. al studied an osteoporosis decision aid in a convenience sample of 20 women and found that knowledge improved from 47% to 83% and decisional conflict was reduced from 50 to 37[17]. In our study, decisional conflict was lower in the decision aid arm than the FRAX/usual care arm, but did not attain statistical significance. However decisional conflict was relatively low in both arms. Our results are also consistent with previous decision aid trials from our group in which we also detected improvements in knowledge, increased patient engagement, and reduced decisional conflict[5,18].

Our study has several strengths. The practices used in our study were usual primary care practices subject to real world time pressures, with usual patients and providers, strengthening the applicability of our results to real-world clinical settings. This was also a patient-level randomized controlled trial that employed adequate randomization, allocation concealment, blinding of data and outcome collectors and data analysts. We used video recording whenever possible to document fidelity and identify and control for contamination, as well as to document the extent to which the intervention, the decision aid, indeed led to shared decision making.

The main limitations of this study, for those who require impact on clinical outcomes to justify investments in shared decision making, are first our inability to determine the impact of this intervention on patient adherence to bisphosphonates, a central issue in osteoporosis care. In our prior trial using a similar tool, we found a significant improvement in the proportion of patients taking bisphosphonates for more than 80% of days prescribed at 6 months [23 (100%) for the Decision Aid arm vs. 14 (74%) for Usual Care arm, p = .009]. In this study, the results were too imprecise to make a judgment. When the two trials are combined in an exploratory meta-analysis, no significant impact is apparent [24 (60%) for the Decision Aid arm vs. 17 (53%) for FRAX/Usual Care arm, p = .96]. Together, these results show no significant effect on adherence with use of the decision aid compared to clinical decision support or usual care across trials, but the sparse data weakens this inference. In addition, clinicians in these two studies had very limited experience in using the decision aid with their patients. We recently showed that clinicians use decision aids as intended only partially and inconsistently, and that the appropriateness of this use was associated with patient knowledge and involvement in the decision making process, thus suggesting an underestimation of the potential efficacy of decision aids when used as intended[19].

The Osteoporosis Choice decision aid improved knowledge and patient engagement and had a high level of patient and clinician satisfaction. Whether this increased patient engagement and involvement translates to higher uptake of bisphosphonates or better adherence remains unclear. Our results suggest this may still be the case, however larger studies are needed to reliably answer this question. To facilitate the exploration of this tool in practice, our group has made freely available an electronic version of the tool for stand-alone use or integrated into the electronic medical record. The tool can be found here: http://osteoporosisdecisionaid.mayoclinic.org

## Conclusion

Compared to usual care with or without the FRAX calculation, use of the Osteoporosis Choice decision aid during the consultation efficiently improves shared decision making about therapy for osteoporosis between primary care clinicians and at-risk women.

## Supporting Information

S1 CONSORT Checklist Consort checklist. (DOC)

S1 Fig Osteoporosis Decision Aid. Example of a filled decision aid.
(TIF)

S1 File Tables A, B, C, D, and E: Sensitivity analysis. Sensitivity analysis comparing decision air arm and FRAX/usual care arm.
(DOC)

S1 Protocol Study protocol. Original study protocol of the Osteoporosis Choice Randomized Trial II.
(DOC)
