# Supplementary figures and images for: Encounter Decision Aid vs. Clinical Decision Support or Usual Care to Support Patient-Centered Treatment Decisions in Osteoporosis: The Osteoporosis Choice Randomized Trial II
Source: PLoS One. 2015 May 26;10(5):e0128063. doi: 10.1371/journal.pone.0128063 (PMC4444262; doi:10.1371/journal.pone.0128063)

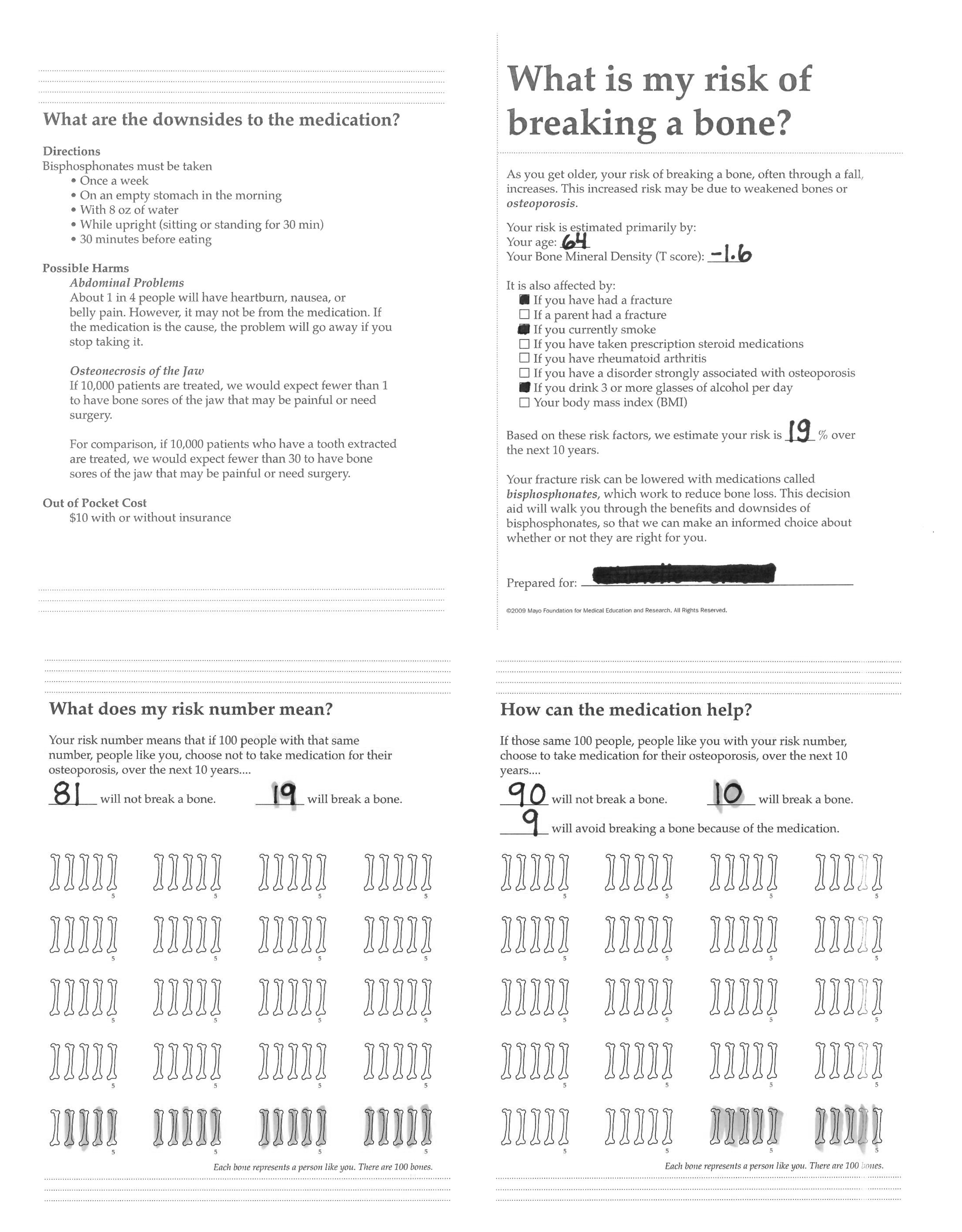

Supplement: S1 Fig — Example of a filled decision aid. (TIF) [file pone.0128063.s002.tif]
