# Supplementary material for: Encounter Decision Aid vs. Clinical Decision Support or Usual Care to Support Patient-Centered Treatment Decisions in Osteoporosis: The Osteoporosis Choice Randomized Trial II
Source: PLoS One. 2015 May 26;10(5):e0128063. doi: 10.1371/journal.pone.0128063 (PMC4444262; doi:10.1371/journal.pone.0128063)
Supplement: S1 File — Sensitivity analysis comparing decision air arm and FRAX/usual care arm. (DOC) [file pone.0128063.s003.doc]

**Comparison of FRAX vs. Usual Care**

Table 1. Patient characteristics

| **Characteristics** | FRAX | Usual Care | P-Value^5^ | P-Value^6^ |
| --- | --- | --- | --- | --- |
| **Patients** | 32 | 13 |  |  |
| Age, y^1^ | 65 (10)  64 (56, 71) | 68 (9)  66 (63, 72) | 0.22 | 0.11 |
| BMI^1^ | 28 (5)  30 (25, 32) | 28 (5)  28 (24, 30) | 0.62 | 0.73 |
| Education^2^ | | | 0.05 | 0.14 |
| High School or Less | 8 (25) | 2 (15) |
| Some College | 14 (44) | 2 (15) |
| 4 yr College + | 7 (22) | 8 (62) |
| Income^2^ (dollars per year) | | | 0.91 | 0.40 |
| <40 000 | 7 (22) | 3 (23) |
| 40-80 000 | 11 (34) | 4 (31) |
| ≥80 000 | 9 (28) | 5 (38) |
| Subjective Numeracy^1^ | 4.4 (0.9) | 4.1 (0.8) | 0.35 | 0.63 |
| Risk of a bone fracture^1^ (FRAX score) | 13 (7)  10 (9, 15) | 11 (5)  9 (8, 13) | 0.36 | 0.55 |
| Low (<10%) | 13 (41) | 8 (62) | 0.50 | 0.49 |
| Moderate (11-20%) | 13 (41) | 4 (31) |
| High (>20%) | 6 (19) | 1 (8) |
| **Clinicians** | 22 | 9 |  |  |
| Gender, female | 12 (55) | 3 (33) | 0.43 | 0.49 |
| Type | | | 0.66 | 0.50 |
| Local Primary Care | 5 (23) | 3 (33) |
| Referral practice | 17 (77) | 6 (67) |
| Clinicians likelihood of prescribing medication^1, 4^ | 2.4 (1.5) | 2.4 (1.3) | 0.69 | 0.66 |
| Encounters^3^ | 1.5, 1 (1, 5) | 1.4, 1 (1, 4) |  |  |

1. Mean (Std. Dev.), Median (IQR)
2. Values missing for patients
3. Mean, Median (Range)
4. Bias assessment of prescribing rates assessed for stratifying patients
5. P-value comparing FRAX vs. Usual care using Wilcoxon rank sum test for continuous outcomes and fisher’s exact test for categorical outcomes.
6. P-value comparing the three arms using Kruskal-Wallis test for continuous outcomes and Fisher’s exact test for categorical outcomes

Table 2: Knowledge and Decisional conflict

|  | FRAX | Usual Care | P-Value | P-Value |
| --- | --- | --- | --- | --- |
| Knowledge^1^ | N=25 | N=11 |  |  |
| Overall | 6 (3, 8) | 3 (2, 10) | 0.73 | 0.25 |
| Items addressed in DA | 4 (2, 5) | 3 (2, 7) | 0.64 | 0.03 |
| Items not addressed in DA | 2 (0, 3) | 0 (0, 3) | 0.71 | 0.89 |
| Knowledge of Risk^2^ | N=28 | N=12 |  |  |
| Risk w/out medication | 9 (32) | 5 (42) | 0.56 | 0.02 |
| Post-treatment risk | 7 (25) | 5 (42) | 0.29 | 0.0002 |
| Decisional Conflict Scale^1^  Overall  Informed subscale  Clarity subscale  Support subscale  Certain subscale  Effectiveness subscale | N=26 | N=10 | 0.66  0.70  0.82  0.53  0.31  0.51 | 0.35  0.30  0.51  0.49  0.31  0.29 |
|  | 17.2 (4.7, 29.7)  25.0 (0, 33)  25.0 (0, 33)  16.7 (0, 33)  8.3 (0, 25)  18.8 (0, 25) | 25.0 (18.8, 28.1)  16.7 (0, 25)  25.0 (16.7, 25)  20.8 (8.3, 25)  25.0 (0, 41.7)  25.0 (0, 25) |  |  |

1– Median (IQR), Wilcoxon Rank sum test p-value for two arm comparison and Kruskal-Wallis test for three arm comparison.

2- Answered correctly (% correct), Chi-square test p-value.

Table 3: Decision and Adherence

|  | FRAX | Usual Care | P-Value^4^ |
| --- | --- | --- | --- |
| Patient decision per survey^1^ | N=27 | N=11 | 0.90 |
| Start Bisphosphonates | 8 (30) | 3 (27) |
| Not Start | 10 (37) | 3 (27) |
| Undecided/Other | 9 (33) | 5 (46) |
| Given prescription during encounter^1^ | 9 (28) | 3 (23) | >0.99 |
| Filled Prescription^1, 2^ | 2 (25) | 2 (100) | 0.13 |
| Adherence: Percentage of days covered^3^ | 85 (81, 89) | 70 (47, 94) | ~ |

1 – Fisher’s exact test p-value.

2- Missing pharmacy records (FRAX=1, usual care=1).

3- Median (Range).

4- p-value comparing FRAX vs. Usual care using Wilcoxon rank sum test for continuous outcomes and fisher’s exact test for categorical outcomes.

Table 4: Involvement, fidelity and satisfaction of patients

|  | FRAX | Usual Care | P-Value | P-Value |
| --- | --- | --- | --- | --- |
| Level of engagement  (OPTION)^1^ | N=19 | N=6 |  |  |
|  | 42.9 (36.1, 49.7) | 41.8 (34.9, 48.8) | 0.66 | 0.34 |
| Fidelity^1^ |  |  |  |  |
|  | 2 (1.4, 2.7) | 1.3 (0.2, 2.5) | 0.36 | <.001 |
| The amount of information received was:^2^ | | | | |
| Just right | 24 (89) | 10 (100%) | 0.55 | 0.74 |
| Not | 3 (11) | 0 |  |  |
| The information received was clear ^2^ | | | | |
| Clear | 18 (69) | 8 (80) | 0.69 | 0.67 |
| Not | 8 (31) | 2 (20) |  |  |
| Helpfulness of Information^2^ | | | | |
| Helpful | 15 (60) | 8 (89) | 0.21 | 0.26 |
| Not | 10 (40) | 1 (11) |  |  |
| Recommend method to others^2^ | | | 0.35 | 0.37 |
| Yes | 20 (77) | 7 (78) |  |  |
| Unsure/No | 6 (23) | 2 (22) |  |  |
| EQOL 5D Health Thermometer^3^ | 82.5 (70, 90) | 88.5 (80, 90) | 0.31 | 0.26 |

1- Mean (95% CI), t-test p-value for two arm comparison and ANOVA for three arm comparison

2- Count (%), Fisher’s Exact test p-value

3-Median (IQR), Wilcoxon rank sum test p-value for two arm comparison and Kruskal-Wallis test p-value for three arm comparison.

Table 5: Clinician satisfaction and decisional conflict

|  | FRAX | Usual Care | P-Value^3^ | P-Value^4^ |
| --- | --- | --- | --- | --- |
| **Satisfaction** | | | | |
| Information was helpful to  the patient^1^ | 12 (44) | 2 (14) | .15 | 0.008 |
| Would recommend method to other providers^1^ | 8 (30) | 4 (31) | >.99 | 0.002 |
| Would present information in the same manner for other treatment decisions^1^ | 12 (46) | 4 (31) | 0.49 | 0.09 |
| **Perception of effectiveness (DCS)**^2^ | 25 (13, 25) | 25 (19, 25) | 0.81 | 0.41 |

Abbreviations: Decisional Conflict Scale DCS.

1. Count (%)
2. Median (IQR)
3. P-value comparing FRAX vs. Usual care using Wilcoxon rank sum test for continuous outcomes and fisher’s exact test for categorical outcomes.
4. P-value comparing the three arms using Kruskal-Wallis test for continuous outcomes and Fisher’s exact test for categorical outcomes
