# Supplementary material for: Encounter Decision Aid vs. Clinical Decision Support or Usual Care to Support Patient-Centered Treatment Decisions in Osteoporosis: The Osteoporosis Choice Randomized Trial II
Source: PLoS One. 2015 May 26;10(5):e0128063. doi: 10.1371/journal.pone.0128063 (PMC4444262; doi:10.1371/journal.pone.0128063)
Supplement: S1 Protocol — Original study protocol of the Osteoporosis Choice Randomized Trial II. (DOC) [file pone.0128063.s004.doc]

**IRB Protocol Template**

**Title:** **Patient participation in decision making with medication adherence in osteoporosis**

**Protocol Version/Date:**  **02-16-2010**

**IRB#:08-006070**

**PI: Victor M. Montori, MD MSc**

**Abstract:**

Bisphosphonates can reduce fracture risk in patients with osteoporosis. Patients may not start and adhere to bisphosphonates, consequently losing quality and length of life. We hypothesize that a decision aid that improves patient-provider communication about fracture risk will improve the quality of treatment decisions: patients will become more involved in the decision-making process and decisions will be more consistent with patients' values and health care goals. We expect this will lead to increased bisphosphonate start and adherence in patients at increased risk of fracture, and that patient with a low risk of fracture will stop or not start bisphosphonates.

To test our hypothesis, we developed OSTEOPOROSIS CHOICE, a decision aid to support the decision to use (or not use) bisphosphonates in postmenopausal women at risk for osteoporotic fractures, based on individualized fracture risk estimates. This practical randomized trial will test the decision aid + risk calculation vs. consultations with only the fracture risk calculator vs. consultations with usual care (no risk calculation, no decision aid). We hypothesize that risk estimation is insufficient to improve quality of decision making and adherence, and both can be enhanced with the full decision aid.

The Normalization Process Analysis (addendum) will enable us to understand the conditions that are necessary to support the implementation / normalization of this decision aid in clinical practice.

The Normalization Process Theory (NPT), developed by Professor Carl May, can inform the evaluation of the processes by which complex interventions, such as decision aids, can become embedded in health care practice. Using this model in the OSTEOPOROSIS CHOICE II study, will enable us to understand the conditions that are necessary to support the implementation / normalization of this decision aid into routine practice. We will focus on the role of the nurse in the outpatient setting, who typically sees the patient prior to the appointment with their primary clinician.

We will also analyze a subset of patient-clinician encounters to identify organizational, patient, and provider characteristics that promote or inhibit uptake of the decision aid in clinical practice.

**Schematic Design of the Study:**

We are conducting a randomized controlled trial (RCT) of a decision aid for female patients with osteopenia or osteoporosis. Patients will be randomized to one of three arms: Intervention (decision aid + FRAX calculator*), FRAX calculator, or Usual care (no FRAX calculation and no decision aid). Clinicians who consent to participate will administer the decision aid to those patients randomized to the intervention.

The Normalization Process Analysis, addendum to Osteoporosis Choice II protocol, is a qualitative assessment.

**The FRAX (Fracture Risk Assessment) tool developed by the World Health Organization is available at: http://www.shef.ac.uk/FRAX/*

**Aims:**

Bisphosphonates can reduce fracture risk in patients with osteoporosis. However, many patients may not start and adhere to bisphosphonates, consequently losing independence, quality, and length of life. Women who are at a low risk for developing an osteoporotic fracture may be unaware of their minimal risk and side effects of treatment, and may be receiving treatment without knowledge of their low risk. We hypothesize that a decision aid that efficiently improves patient education and communication with their provider about fracture risk, and about using bisphosphonates to reduce that risk, will improve the quality of treatment decisions. We anticipate that patients will become more involved in the decision-making process and decisions will be more consistent with patient's values and health care goals. We expect this will lead to increased bisphosphonate start and adherence in patients at high risk of fracture, and to patient's stopping medication or not starting in patients at low risk of fracture.

Normalization Process Analysis Aim: at the end of the study, we will have results concerning the efficacy of the decision aid from the trial, as well as evidence of the extent to which nurses can facilitate the normalization of the decision aid into clinical practice.

Analysis of video-taped patient-clinician encounters: as a secondary aim, we will analyze videotapes of patient-clinician encounters to identify organizational, patient, and provider characteristics that promote or inhibit uptake of the decision aid in clinical practice.

**Methods**

**Description of Recruitment Methods:**

The Olmsted Medical Center (OMC), Rochester, has been added as a site. OMC study coordinators will recruit and consent patients. Enrolled patients and clinicians who consent to having the clinical visit recorded will be video and/or audio recorded via the FLIP cameras. Participating clinicians and patients will complete surveys following each encounter. The OMC study coordinator will notify the Mayo Clinic study coordinator each time a patient has enrolled in the study and arrange for all data (surveys, case report forms) and the FLIP camera to be hand delivered to the Mayo Clinic study coordinator.

In the event that there are any modifications in consent documents, the Mayo Clinic Study Coordinator will submit modifications to the OMC IRB, and provide revisions to the OMC Study Coordinator.

The coordinating center PI (Dr. Victor Montori) has all contact information for OMC personnel, and will communicate / ensure that OMC has the most current version of the protocol and any amendments to the protocol.

Any deviations from the protocol by OMC will be communicated to the Mayo Clinic study coordinator or PI within 3 business days.

**Data management, video and audio recordings between OMC and Mayo clinics:**

Both sites will be required to use the current version of all documents and forms. All forms, documents and video/audio recordings completed at OMC will be immediately brought to the OMC Research Office. The OMC staff will contact a Mayo, KER Unit study coordinator, who will arrange to pick up the study materials that day at the OMC Research Office. If this cannot be accomplished that day, the study materials will be placed in a locked cabinet until the Mayo study coordinator is available. The Mayo Study Coordinator will place the "Flip" camera and all study documents in a locked PHI folder (which is HIPAA compliant). The study materials will then be immediately transported to the KER Unit office, where the all study materials will be stored in a locked file cabinet. Digital recordings from the FLIP camera are uploaded to the KER Unit's secure server, and deleted from the portable devices. The video and audio files are identified using a code number that does not include the name of the clinician or patient. Transcriptions will omit patient and clinician names that may have been stated during the recording. The research data are only accessible with password protected and logged access at the KER Unit at Mayo Clinic. All Mayo personnel with access to study data have received human subjects’ research training.

**How will patients be identified?**

We will identify eligible women from lists of upcoming bone mineral density (BMD) evaluations and from participating providers' appointment calendars.

For the Normalization Process Analysis, we will identify 3 nurses who work directly with one primary care clinician, and meet with them to determine the following:

Qualitative Taxonomy of Tasks: This group of nurses will be provided with background information concerning the OSTEOPOROSIS CHOICE II decision aid study, and then consider the work that goes into the preparation of the decision aid, and a preliminary discussion with the patient of the patient’s risk factors for an osteoporotic fracture in the next 10 years. They will focus on the work of the nurse, as well as the other members of their work unit (other nurses, clinicians, clerks). Participants will build a taxonomy of tasks involved in making the intervention a part of their routine, qualitatively assess the relative importance to participants, and identify potential conflicts between new and existing tasks.

Verifiable Propositions: We will subject the above data to qualitative procedures of constant comparison (Glaser 1965) to develop a master taxonomy of tasks, subjective assessments of their relative importance, and potential conflicts between proposed and existing tasks. This master taxonomy will be used to identify factors that will promote or inhibit the operationalization of the intervention, and a set of theory-based explanations for these factors can be empirically tested.

Structured Data: The verifiable propositions will: a) be distilled as additional questions for post-visit surveys of nurse(s) (e.g., “How difficult was it for you to prepare the decision and discuss the patient’s risk factors, given the other roles and responsibilities you hold?), and b) provide a framework for a structured observation manual. Study coordinators will carry this observational manual during the study, and make notes of verbal, nonverbal and other clues that indicate difficulties faced by nurses or other unit personnel, when nurses are preparing / discussing the decision aid with a patient.

Normalization Process Analysis: After all patients have been recruited, we will meet with participating nurses to determine the work that actually went into the implementation of the decision aid prep and discussion, what work this represented to others, and other factors that may inhibit or facilitate implementation.

When a patient has consented to participate in the study, the 3 identified nurses will meet with their patient and do the following:

After the patient has met with the study coordinator and provided informed consent to participate, the clinician will meet with their patient as they usually do in the examination room. If the clinician and their patient agree to do so, their visit will be videotaped. The consent document for clinician will have the following language:

“Your patient will be randomized to one of three groups by chance. Depending on which group your patient is in, you may share information about your patient’s treatment options, including bisphosphonate therapy, using different visual aids. If you and your patient agree to do so, your visit with your patient will be videotaped. If your patient is randomized to the control arm, you will meet with your patient in the usual fashion. If your patient is randomized to the FRAX or the FRAX + decision aid arm, you will discuss their fracture risk with them. Following your visit with your patient you will be asked to answer a short survey of 3-4 questions, which should take about one minute to complete.”

**How will patients be contacted?**

We will request that participating clinicians schedule an appointment to discuss abnormal BMD results with the patient during an office visit. For patients with a scheduled appointment, we will approach the patient after they have been roomed for this appointment, prior to the discussion with their clinician. The study coordinator will inform patients about the study, will confirm eligibility, and obtain written informed consent in a private room. When there is insufficient time to consent a scheduled patient prior to the appointment time, the study coordinator or clinical assistant may contact the patient by phone and request the patient arrive 15 minutes early to the appointment.

**Recruitment Materials (if applicable):**

Clinicians will be contacted by e-mail to inform them of their patient's eligibility for the study and request permission for us to approach them.

**Patient Population:**

**Number (total, each subgroup)** 150, 50, 50, and 50

We expect that 30 patients will take part at Olmsted Medical Center, Rochester

**Gender:**

Male:

Female: X

Ages: 50 to 90 years

**Clinician Population:**

Number (max. total): 100

Male: X

Female: X

Ages: > 21 years of age

**Inclusion Criteria:**

- 1. Peri/ post menopausal Caucasian, Black, Hispanic and Asian women aged 50-90 with a T-score < -1.0.
  2. Identified by their providers as potentially eligible for treatment with a bisphosphonate.
  3. Are available for phone follow-up 6 months after randomization.
  4. Patients with FRAX <20% risk who have taken a bisphosphonate for < 5 years.

**Exclusion Criteria:**

1) Patients with FRAX >20% risk who are taking bisphosphonates.

2) Have major learning barriers (in their provider's judgment), such as hearing impairment, or dementia, that would compromise their ability to give written informed consent

3) Are non-English speaking

**Step-by-Step Schedule (include all procedures, therapies – attach a table or flow chart if there are multiple procedures or visits and indicate the window of number of days in which the participant may return for follow-up visits):**

See attachment (Protocol Steps - Trial Flowsheet)

**Biospecimen (types, number, volume, processing, storage):** Not applicable

**Plan for Dose Modification if Toxicity occurs (if applicable):** Not applicable

**Statistical Considerations**

**Endpoints**

**Primary:** Medication start/stop, knowledge, and patient involvement will be the primary outcome measures. Medication start / stop will be assessed at meeting with clinician and at 6 months via self-report and pharmacy records.

**Secondary:** Decisional quality (i.e. knowledge, decisional conflict) will be assessed at baseline and at 6 months via phone surveys.

Normalization Process Analysis: At the end of the study, we will have results concerning the efficacy of the decision aid from the trial, as well as evidence of the extent to which nurses can facilitate the normalization of the decision aid into clinical practice.

Secondary analysis of videotaped patient clinician encounters. Two reviewers working independently and in duplicate will review the video recordings to determine eligibility and to extract data for analyses. Videotapes of eligible encounters will be reviewed to identify characteristics of patients, clinicians and patient-clinician interactions associated with use of the decision aid in clinical practice after the trial is complete.

**Power Statement:**

We plan to enroll and randomize 150 eligible patients. While this number of patients is insufficient to reach a definitive answer about the efficacy of our decision aid and its' effects on adherence, it is sufficient to ascertain meaningful differences in tool acceptability and knowledge gains as well as inform a plausible effect size in relation to its impact on adherence. Thus, we will be powered (80%) to detect a difference (alpha = 0.05) in adherence rates at 6 months of 30%. While a minimally important increase in adherence has not been reported, a 10% increase (e.g. from 65% to 75%) appears as the smallest difference that could justify the use of decision aids in practice. We will pool patients from the first pilot (with the exception of the low risk patients on bisphosphonates at time of recruitment), which will generate 95% confidence intervals with a precision of 17%, or a 90% confidence interval (akin to a one-sided hypothesis test) of 14% (44). This means that we have enough power to abandon plans for a multicenter trial when the point estimate of our trial indicates a decrease in adherence because of the use of the decision aid of greater than or equal to 7% or greater than or equal to 4% respectively.

**Data Analysis:**

We will use hierarchical models (that take into account clustering/contamination) to estimate the absolute difference in bisphosphonates initiation and adherence rates between groups and the associated 95% confidence interval. We will use similar analyses for other dichotomous outcomes (e.g., adherence). We will use generalized estimation equations to compare the change in intermediate continuous outcomes (decisional conflict, knowledge) by arm after adjusting for clustering across physicians. With the exception of the low risk group on treatment, an analysis of Osteo II data may be combined with the Osteo I Choice study.

**Human Safety Aspects**

**Risks**: This is a minimal risk protocol.

**Individual Patient Stopping Rules** (if applicable):

**DSMB** (if applicable):

Members: Not applicable

Charter: Not applicable

**Stopping Rules for Efficacy and** Safety (if applicable): Not applicable

**Questionnaires that ask about Depression** (if applicable)

Included in study? Yes  No

If “Yes”, state the plan of management for subjects with possible depression:

When submitting this form to IRBe, attach a copy of the budget and consent form. Thank you.
